# Joint Design of Space-Time Transmit and Receive Weights for Colocated MIMO Radar

**DOI:** 10.3390/s18082722

**Published:** 2018-08-18

**Authors:** Ze Yu, Shusen Wang, Wei Liu, Chunsheng Li

**Affiliations:** 1 School of Electronic and Information Engineering, Beihang University, Beijing 100083, China; shusenwang@buaa.edu.cn (S.W.); 00178@buaa.edu.cn (C.L.); 2 Department of Electronic & Electrical Engineering, University of Sheffield, Sheffield S1 4ET, UK; w.liu@sheffield.ac.uk

**Keywords:** colocated multiple-input multiple-output (MIMO) radar, space-time weights, closed-loop system

## Abstract

Compared with single-input multiple-output (SIMO) radar, colocated multiple-input multiple-output (MIMO) radar can detect moving targets better by adopting waveform diversity. When the colocated MIMO radar transmits a set of orthogonal waveforms, the transmit weights are usually set equal to one, and the receive weights are adaptively adjusted to suppress clutter based on space-time adaptive processing technology. This paper proposes the joint design of space-time transmit and receive weights for colocated MIMO radar. The approach is based on the premise that all possible moving targets are detected by setting a lower threshold. In each direction where there may be moving targets, the space-time transmit and receive weights can be iteratively updated by using the proposed approach to improve the output signal-to-interference-plus-noise ratio (SINR), which is helpful to improve the precision of target detection. Simulation results demonstrate that the proposed method improves the output SINR by greater than 13 dB.

## 1. Introduction

For colocated multiple-input multiple-output (MIMO) radar, the antennas are close to each other, and each antenna serves as a transceiver. Compared with single-input multiple-output (SIMO) radar, colocated MIMO radar can achieve better detection of moving targets by adopting waveform diversity [1,2,3].

To improve the performance of clutter mitigation, orthogonal waveforms are widely used in colocated MIMO radar [4,5]. Each transceiver transmits one unique and orthogonal waveform, and then receives and separates all the return echoes by using the appropriate matched filter bank. Compared with SIMO radar, more virtual phase centers are formed, and more degrees of freedom (DOFs) are achieved. Combined with space-time adaptive processing (STAP), MIMO radar with orthogonal waveforms can obtain better clutter mitigation performance and identify more target parameters. To realize good correlation properties of the transmitted signals, the common solution is to solve an optimization problem with constraints. The existing orthogonal waveform design algorithms can be divided into two categories. The first, which includes Cross Entropy [6], simulated annealing [7], and the Genetic Algorithm algorithms [8,9], optimizes the polyphase code sequences to achieve low aperiodic auto-correlation sidelobe peaks and cross-correlation peaks. The second, which includes the efficient cyclic algorithm [10] and the monotonic minimizer for integrated sidelobe level [11] and the coordinate-descent algorithm [12], optimizes the unimodular sequences to achieve a good integrated or peak sidelobe level of the auto-correlation function.

Although they provide more DOFs, orthogonal waveforms induce the omnidirectionality of the antenna, which reduces the signal-to-noise ratio (SNR). One solution is to adopt beamforming techniques [13,14,15,16,17], which design partial coherent transmitted signals by optimizing the signal covariance matrix with the constraint of the desired beampattern. In order to improve the signal-to-interference-plus-noise ratio (SINR), the joint design of the transmit signal and receive filter has been proposed [18,19,20,21,22]. In [18], constrained optimization procedures are devised based on phase-only waveforms to sequentially improve the SINR. In [19], the priori knowledge about geographical information is used in the optimization. In [20], it is assumed the target Doppler frequency is unknown and the worst-case SINR at the output of the filter bank is considered in the robust joint design. For non-cooperative radar networks, design of coded coherent waveforms has been presented to improve the SINR of each active radar by resorting to the theory of potential games [21]. For MIMO radar, the design of a space-time transmit code and space-time receive filter has been presented to improve the worst case SINR by optimizing the coded coherent signals [22]. For colocated MIMO radar, transmitted waveform covariance matrix has been optimized to focus the transmit beampattern into the target direction to improve the SINR [23]. The design of a space-time transmit code and receive filter has been presented to improve the SINR by optimizing the partial coherent signals under different constraints, such as a similarity constraint and a constant modulus requirement [24], integrated sidelobe level and peak sidelobe level constraints at the pulse compression output [25], or for generating BPSK waveforms [26].

The existing literature about improving the output SINR for colocated MIMO radar focuses on optimizing the covariance matrix of partially coherent signals, which reduces the dimension of the measurement space and results in the loss of abilities to suppress high-rank clutter. To preserve the full spatial degrees of freedom and improve the output SINR, a joint design of transmit and receive weights is proposed for colocated MIMO radar using orthogonal waveforms, and an iterative framework is determined to update the weights in real time. Initially, all possible moving targets are detected by setting a lower threshold, which may cause high false alarm rate. Then in each direction where there may be moving targets, the space-time transmit and receive weights are iteratively updated by exploiting the clutter covariance matrix and moving target status including locations and velocities. Through iteration, the output SINR is gradually increased, which is beneficial to improve detection performance and reduce the false alarm rate.

This paper is structured as follows. Section 2 introduces the signal model for colocated MIMO radar, and illustrates the optimization problem with constraints on the transmit and receive weights for improving the output SINR. Section 3 presents the joint design method of space-time transmit and receive weights. By exploiting the information about the dynamic environment contained in the return echoes, the transmit-receive weights can be updated in real time. The simulation results are demonstrated and analyzed in Section 4, and Section 5 concludes the paper.

## 2. Optimization Model for Colocated MIMO Radar

**Figure 1:**
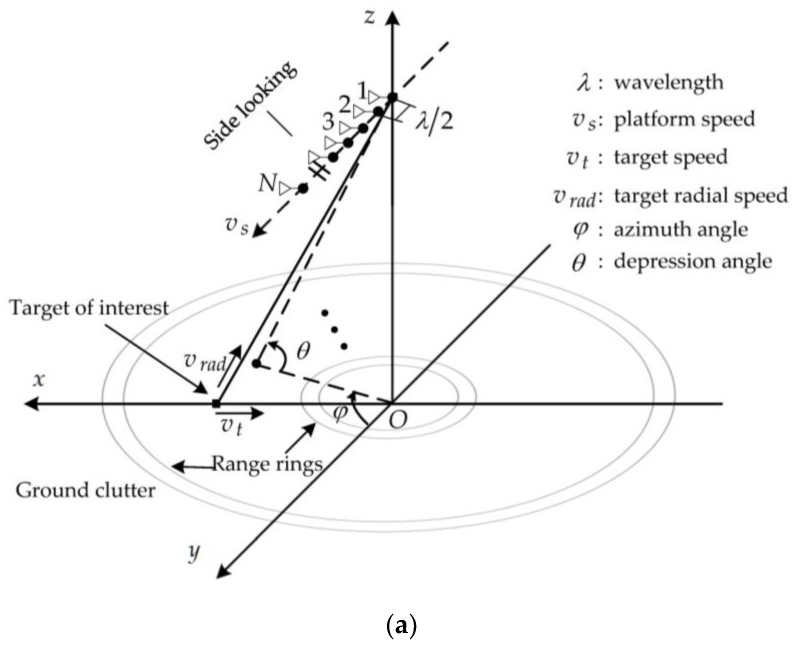

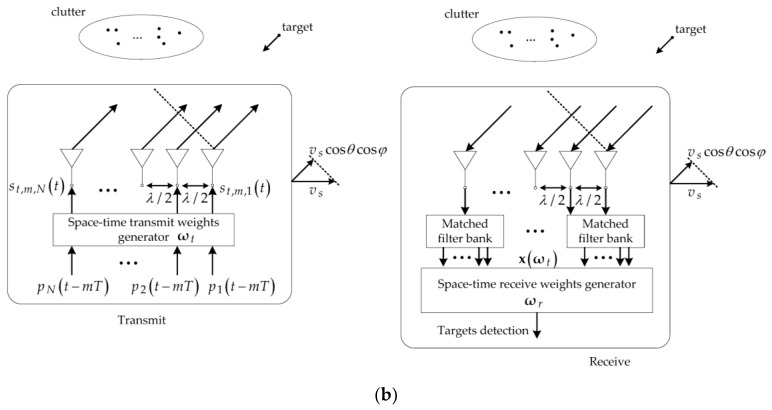
Illustration of the multiple-input multiple-output (MIMO) radar with uniform linear arrays. (**a**) shows the observation geometry, and (**b**) describes the transmit and receive processes.

Figure 1a shows the observation geometry. A right-handed Cartesian coordinate system is established, where the origin is located at the center of the observed area and the *x*-axis, *y*-axis, and *z*-axis represent the broadside direction, flight direction, and height direction, respectively. The radar moves along the *y*-axis. A uniform linear array with N identical antennas is mounted on the radar. The spacing between the two adjacent antennas is half of a wavelength. A target moves at a speed of $v_{t}$, and $v_{rad}$ denotes the radial component of $v_{t}$. $v_{s}$ denotes the platform speed. φ and θ are the azimuth and depression angles, respectively. In this paper, “speed” is loosely used to mean “magnitude of the velocity vector”. According to [27], angles and speeds are defined as follows:

- Platform speed: This is the speed of the platform along the flight path.
- Target speed: This is the speed of one moving target along the moving direction, which can be any direction in the free space.
- Target radial speed: This is the speed of one moving target along the line-of-sight direction.
- Nadir: The nadir is the point on the area directly below the radar phase center, so that the “normal” to the area at the nadir passes through the radar phase center.
- Radar track: As the nadir point moves along the area, it traces out the radar track.
- Depression angle: This is the remaining angle of the off-line angle, which is the angle from the target to the nadir relative to the radar phase center.
- Azimuth angle: This is the clockwise angle from the direction of the radar track to the direction of interest relative to the nadir point viewed from above (i.e., projected to the ground plane).

Usually in radar systems, the echoes corresponding to a group of multiple pulses are coherently processed, and a declaration is made whether targets exist or not. The duration of these multiple pulses is called the coherent processing interval (CPI). Figure 1b describes the transmit and receive processes. In each CPI, each antenna transmits the orthogonal pulse train consisting of M rectangular pulses, which are $p_{n}\left(t-mT\right),m=1,2,\cdots ,M$, where T denotes the pulse repetition interval, n=1,2,⋯,N represents the *n*-th antenna, and

(1) $$\int_{-T_{p}/2}^{T_{p}/2}p_{i}\left(t\right)p_{j}^{*}\left(t\right)dt=\{\begin{array}{cc} 1 & i=j\\ 0 & else \end{array}\;i=1,\cdots ,N;\;j=1,\cdots ,N$$

where $T_{p}$ is the pulse width and ${\left(\cdot \right)}^{*}$ denotes the conjugate operator. At the *m*-th transmission moment, all the pulses are weighted by the space time transmit weights $\omega_{t}\left(m,n\right)$ to form the signal $s_{t,m,n}\left(t\right)$ which is radiated in the free space. $s_{t,m,n}\left(t\right)$ equals

(2) $$s_{t,m,n}\left(t\right)=\omega_{t}\left(m,n\right)p_{n}\left(t-mT\right)\exp\left[j2\pi f_{c}\left(t-mT\right)\right]$$

where $f_{c}$ is the carrier frequency, all the space time transmit weights $\omega_{t}\left(m,n\right)$ will form the space time transmit weight vector

$$\omega_{t}={\left[\omega_{t}\left(1,1\right),\omega_{t}\left(2,1\right),\cdots ,\omega_{t}\left(M,1\right),\omega_{t}\left(1,2\right),\omega_{t}\left(2,2\right),\cdots ,\omega_{t}\left(M,N\right)\right]}^{T}$$

The return echoes reflected from the clutters and moving target are received by each antenna and pass through the matched filter bank. The filter bank is composed of N different matched filters, and each filter corresponds to one transmit waveform. Thus, the echoes are separated. By applying the space-time receive weights $\omega_{r}$, the clutter is adaptively suppressed and moving targets are detected.

To focus on the optimization of transmit-receive weights, the simplified assumptions are adopted in the paper, which are not ambiguous returns as well as not returns from adjacent range cells. After demodulation and matched filtering, the target’s echo and the clutter’s echoes received by the *n*-th antenna transmitted by the *i*-th antenna at the *m*-th pulse repetition interval can be expressed as

(3) $$\begin{array}{cc} x_{t,m,i,n}\left(\omega_{t}\right) & =\sum_{k=1}^{K}A_{k}^{\left(t\right)}\omega_{t}\left(m,i\right)\exp\left[j\frac{2\pi }{\lambda }\left(m-1\right)T\left(2v_{s}\cos\varphi_{k}^{\left(t\right)}\cos\theta +2v_{rad,k}\right)\right]\\ & \times \exp\left[j\frac{2\pi }{\lambda }\left(\left(i-1\right)\frac{\lambda }{2}\cos\theta \cos\varphi_{k}^{\left(t\right)}+\left(n-1\right)\frac{\lambda }{2}\cos\theta \cos\varphi_{k}^{\left(t\right)}\right)\right],\\ & \begin{array}{ccc} m=1,\cdots ,M & i,n=1,\cdots ,N & \end{array} \end{array}$$

and

(4) $$\begin{array}{cc} x_{c,m,i,n}\left(\omega_{t}\right) & =\int_{\varphi =0}^{2\pi }A^{\left(c\right)}\left(\varphi \right)\omega_{t}\left(m,i\right)\exp\left[j\frac{2\pi }{\lambda }\left(m-1\right)T\left(2v_{s}\cos\varphi \cos\theta \right)\right]\\ & \times \exp\left[j\frac{2\pi }{\lambda }\left(\left(i-1\right)\frac{\lambda }{2}\cos\theta \cos\varphi +\left(n-1\right)\frac{\lambda }{2}\cos\theta \cos\varphi \right)\right]d\varphi ,\\ & \begin{array}{cc} m=1,\cdots ,M & i,n=1,\cdots ,N \end{array} \end{array}$$

As there is a one-to-one functional mapping relationship between the depression angle θ and some range gate [12], and each range gate is a minimum range sampling unit, the independence of return echoes on θ has been omitted to simplify the expressions. The vector format of the target’s echo and clutter’s echoes can be expressed as

$$\begin{array}{l} x_{t}\left(\omega_{t}\right)={\left[x_{t,1,1,1},x_{t,2,1,1},\cdots ,x_{t,M,1,1},x_{t,1,2,1},x_{t,2,2,1},\cdots ,x_{t,M,N,N}\right]}^{T}\\ x_{c}\left(\omega_{t}\right)={\left[x_{c,1,1,1},x_{c,2,1,1},\cdots ,x_{c,M,1,1},x_{c,1,2,1},x_{c,2,2,1},\cdots ,x_{c,M,N,N}\right]}^{T} \end{array}$$

In Equations (3) and (4), $\omega_{t}={\left[\omega_{t}\left(1,1\right),\omega_{t}\left(1,2\right),\cdots ,\omega_{t}\left(M,N\right)\right]}^{T}$, K is the number of targets and equals to 1 in this paper, λ is the wavelength, $v_{s}$ is the platform speed, θ is the depression angle, i denotes the *i*-th transmit antenna, and n denotes the *n*-th receive antenna. $A_{k}^{\left(t\right)}$, $v_{rad,k}$ and $\varphi_{k}^{\left(t\right)}$ are the complex amplitude, radial velocity, and relative azimuth angle of the *k*-th target, respectively. $A_{}^{\left(c\right)}\left(\varphi \right)$ is the clutter complex amplitude and is assumed to be characterized by a circularly symmetric complex Gaussian distribution [28,29].

In the temporal domain, the angular frequency is a reflection of the rate of phase change. Its counterpart in spatial domain can be defined similarly [30]. The normalized spatial frequency is a reflection of the rate of phase change with the spatial antennas in Hertz, and can be defined as $f_{s}=d\frac{\cos\theta \cos\varphi }{\lambda }$, where d is the spacing between two adjacent antennas which is half of a wavelength in this paper, therefore the normalized spatial frequency is $f_{s}=\frac{\cos\theta \cos\varphi }{2}$. Let $s_{sf}$ and $s_{td}$ denote the space steering vector and time steering vector, which represent the spatial phase and temporal phase in the return echoes, respectively. They are defined as

(5) $$s_{sf}={\left[\begin{array}{cccc} 1 & \exp\left(j2\pi f_{s}\right) & \cdots & \exp\left(j2\pi f_{s}\left(N-1\right)\right) \end{array}\right]}_{}^{T}$$

and

(6) $$s_{td}={\left[\begin{array}{cccc} 1 & \exp\left(j2\pi f_{d}\right) & \cdots & \exp\left(j2\pi f_{d}\left(M-1\right)\right) \end{array}\right]}_{}^{T}$$

where $f_{s}$ and $f_{d}$ denote the normalized spatial frequency and the normalized Doppler frequency, respectively, and ${\left(\cdot \right)}^{Τ}$ denotes the transpose operator.

Define $s_{t}=s_{sf}⊗s_{td}$ and $s_{r}\left(\omega_{t}\right)=s_{sf}⊗\left(\omega_{t}⊙s_{t}\right)$ as the transmit and receive space-time steering vectors, respectively, where ⊙ is the Hadamard product and ⊗ is the Kronecker product. $s_{t}$ represents the unweighted echo phase term vector for the transmit. $s_{r}\left(\omega_{t}\right)$ represents the weighted echo reflected by one target whose reflection amplitude is one. Therefore, the echo from one range gate consisting of moving targets is expressed as

(7) $$x\left(\omega_{t}\right)=\sum_{k=1}^{K}A_{k}^{\left(t\right)}s_{r}^{\left(t,k\right)}\left(\omega_{t}\right)+n+\int_{0}^{2\pi }A_{}^{\left(c\right)}s_{r}^{\left(c\right)}\left(\varphi ,\omega_{t}\right)d\varphi \;$$

where n is the vector of circularly symmetric white Gaussian noise samples with zero-mean and covariance matrix $\sigma_{n}^{2}$ [25], and $s_{r}^{\left(t,k\right)}\left(\omega_{t}\right)$ and $s_{r}^{\left(c\right)}\left(\varphi ,\omega_{t}\right)$ are the receive space-time steering vectors for the *k*-th target and clutter, respectively.

To improve the SINR, the interference-plus-noise power after receive weighting should be minimized under the condition that the target power is preserved. Therefore, the following optimization model should be satisfied:

(8) $$\underset{\omega_{t},\omega_{r}}{\min}\omega_{r}^{H}Q_{q}\left(\omega_{t}\right)\omega_{r},\;\begin{array}{c} \begin{array}{cc} s.t. & \omega_{r}^{H}s_{r}\left(\omega_{t}\right)=1 \end{array}\\ \begin{array}{cc} & \omega_{t}^{H}s_{t}=g \end{array} \end{array},$$

where

(9) $$Q_{q}\left(\omega_{t}\right)=E\left[\left(n+\int_{0}^{2\pi }A_{}^{\left(c\right)}s_{r}^{\left(c\right)}\left(\varphi ,\omega_{t}\right)d\varphi \right){\left(n+\int_{0}^{2\pi }A_{}^{\left(c\right)}s_{r}^{\left(c\right)}\left(\varphi ,\omega_{t}\right)d\varphi \right)}^{H}\right],$$

E[⋅] and ${\left(\cdot \right)}^{H}$ are the expectation and the conjugate transpose operators, respectively, and $\omega_{r}$ denotes the space-time receive weights. $g=1_{MN}^{H}s_{t}$, where $1_{MN}$ is a column vector of all-ones with the dimension of MN. When radar starts to illuminate some area, there is no target information as well as clutter information, then transmit weights will be set to 1 to achieve the original clutter suppression ability. *g* is to keep the radiation power reaching the target constant, and will hold constant during the illumination time as $g=1_{MN}^{H}s_{t}^{☆}$,where $s_{t}^{☆}$ indicates the target’s direction where the received radiation power will keep constant.

The objective function in Equation (8) is meant to minimize the interference-plus-noise output power. The first constraint indicates that the target power after receive weighting remains constant and the second constraint guarantees that the radiation power reaching the target is also constant. The space-time transmit and receive weights will be jointly and adaptively designed to satisfy Equation (8).

## 3. Joint Design of Transmit and Receive Weights

**Figure 2:**
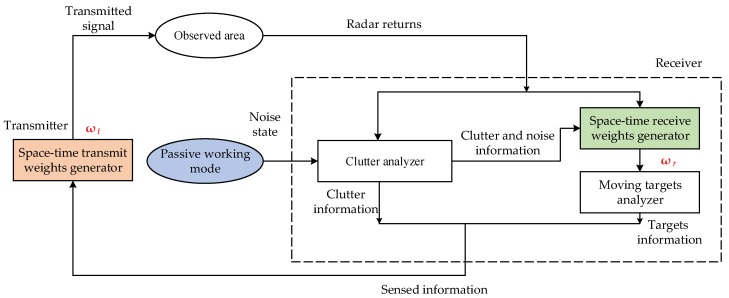
Joint design of space-time transmit and receive weights.

Joint design of transmit and receive weights is implemented iteratively, which is illustrated in Figure 2. Before starting to illuminate the observation area, the radar system works in the passive mode to records noise data [31,32]. And the noise covariance matrix can be obtained as

(10) $$Q_{n}=\frac{1}{L}\sum_{l=1}^{L}n_{l}n_{l}{}^{H}.$$

where $n_{l}$ represents the space-time data of the l-th range gate, and *L* is the number of range gates [12]. The noise covariance matrix is assumed to be unchanged during illumination.

In the first CPI, the transmitter sends out orthogonal waveforms weighted by the initial space-time transmit weights (STTWs) which are set to all ones. The clutter analyzer acquires the clutter pulse noise covariance matrix by processing the return echoes. If the echo in the $l_{0}$-th range gate is processed, the clutter-plus-noise covariance matrix can be obtained by analyzing echoes in other range gates

(11) $$Q_{q}=\frac{1}{L-1}\sum_{l=1,l\neq l_{0}}^{L}x_{l}x_{l}{}^{H}.$$

where $x_{l}$ represents the echo in the l-th range gate. Since the clutter and noise are statistically independent, the clutter covariance matrix can be achieved $Q_{c}=Q_{q}-Q_{n}$.

The space-time-receive weights generator optimally designs the space-time receive weights (STRWs) based on the space time adaptive technology [33,34]. In the moving targets analyzer, the echoes are weighted by the STRWs to suppress the clutter, and a lower threshold is set to detect moving targets. Although the lower threshold results in a higher false alarm rate, it is guaranteed that no targets are missed. Then the locations and velocities of each possible target are extracted.

In the subsequent CPIs, the system works in a closed-loop mode. For one direction where there may be a target of interest, the STTWs and STRWs are adaptively updated by applying the proposed approach. From one CPI to the next CPI, the interference-plus-noise output power is suppressed more and more, which leads to an improvement of the SINR. The moving targets analyzer raises the threshold with the SINR to better extract the information of the target. When the difference of SINRs between adjacent CPIs is little, another direction is selected to perform the closed-loop.

If the information about clutter and target is known to the system designer, the optimization problem about the transmit-receive weights in Equation (8) will have optimal solutions, since the optimization problem is a quadratic objective function with two linear constraints whose hessian matrix is positive definite [35]. If the statistic characteristics of clutter is stationary and the target keeps the same motion state, the proposed method will converge, which is guaranteed by the fact that the optimization problem listed in Equation (8) has an optimal solution. The transmit-receive weights are designed when the proposed iterative method converges and will be the optimal solution mentioned above.

In the joint design, the STRWs are calculated based on the deterministic STTWs. Therefore, Equation (8) can be simplified as

(12) $$\underset{\omega_{r}}{\min}\omega_{r}^{H}Q_{q}\omega_{r},\;\begin{array}{cc} s.t. & \omega_{r}^{H}s_{r}=1 \end{array},$$

where only STRWs remain in the constraint. The STRWs can be obtained by applying the linear weighting as [34]

(13) $$\omega_{r}=\frac{Q_{q}^{-1}s_{r}}{s_{r}^{H}Q_{q}^{-1}s_{r}}.$$

By solving the optimization problem (8) based on the Lagrange multiplier method (see Appendix A), the optimal STRWs can be obtained

(14) $$\omega_{t}^{}={\left(\frac{\left[\left(D_{22}-gD_{12}\right)Q_{c,r}^{-1}\left(1_{MN}\right)s_{1}+\left(gD_{11}-D_{21}\right)Q_{c,r}^{-1}\left(1_{MN}\right)s_{2}\right]}{D_{11}D_{22}-D_{12}D_{21}}\right)}_{}^{*},$$

where

$$s_{1}=\left[\left(\sum_{i=1}^{N}s_{sf,i}\omega_{r,i}^{*}\right)⊙s_{t}^{}\right]$$

$$s_{2}=s_{t}^{*}$$

and

(15) $$\begin{array}{l} D_{11}=s_{1}^{H}Q_{c,r}^{-1}\left(1_{MN}\right)s_{1}\\ D_{12}=s_{1}^{H}Q_{c,r}^{-1}\left(1_{MN}\right)s_{2}\\ D_{21}=s_{2}^{H}Q_{c,r}^{-1}\left(1_{MN}\right)s_{1}\\ D_{22}=s_{2}^{H}Q_{c,r}^{-1}\left(1_{MN}\right)s_{2} \end{array}.$$

In Equations (14) and (15), $s_{sf,i}^{}=\exp\left(j2\pi f_{s}\left(i-1\right)\right)$ represents the *i*-th element of the space steering vector; $\omega_{r,i}^{}$ represents the *i*-th sub vector of the STRWs vector $\omega_{r}={\left[\omega_{r,1}^{Τ},\omega_{r,2}^{Τ},\cdots ,\omega_{r,N}^{Τ}\right]}^{Τ}$; $Q_{c,r}\left(1_{MN}\right)=FQ_{c}\left(1_{MN}\right)F^{H}$, where $F=\left[Diag\left\{\omega_{r,1}^{*}\right\},Diag\left\{\omega_{r,2}^{*}\right\},\cdots ,Diag\left\{\omega_{r,N}^{*}\right\}\right]$, and Diag{a} denotes the diagonal matrix whose diagonal elements are the elements of vector a; and the clutter covariance matrix $Q_{c}\left(1_{MN}\right)$ equals

(16) $$Q_{c}\left(1_{MN}\right)=Q_{q}\left(1_{MN}\right)-Q_{n},$$

where $Q_{q}\left(1_{MN}\right)$ and $Q_{n}$ denote the clutter pulse noise covariance matrix and the noise covariance matrix, respectively. $Q_{q}\left(1_{MN}\right)$ can be estimated from the secondary data which contains only clutter and noise [36]. $Q_{n}$ can be estimated from the collected noise data when the radar system works in the passive mode.

## 4. Simulation and Analysis

### 4.1. Simulation Parameters

Simulation parameters are listed in Table 1, which refer to the standard parameters set in [34]. The radar platform flies parallel to the ground at a uniform speed, while the target moves perpendicular to the flight direction, as shown in Figure 1. Additionally, four orthogonal transmit waveforms generated by the genetic algorithm [8] and corresponding receive filters are used in the simulation. The true interference and target parameters are assumed perfectly known in the simulation results. To valid the proposed method, the conventional method serves as a standard performance where the STTWs are all set equal to one and STTWs are optimized by the space-time adaptive technology.

### 4.2. Simulation Results and Analysis

This paper adopts orthogonal transmit waveforms to preserve high DOFs and joint design of transmit-receive weights to improve the output SINR. Here, the improvement factor (IF) is used to evaluate the performance of the colocated MIMO radar and is defined as the ratio of the output SINR to the input SINR [34], i.e.,

(17) $$\begin{array}{cc} IF & =\frac{\omega_{r}^{H}\left(f_{ST},f_{SR},f_{D}\right)s_{r}\left(f_{ST},f_{SR},f_{D}\right)s_{r}^{H}\left(f_{ST},f_{SR},f_{D}\right)\omega_{r}\left(f_{ST},f_{SR},f_{D}\right)}{\omega_{r}^{H}\left(f_{ST},f_{SR},f_{D}\right)Q_{q}\omega_{r}\left(f_{ST},f_{SR},f_{D}\right)}\\ & \times \frac{tr\left(Q_{q}\right)}{s_{r}^{H}\left(f_{ST},f_{SR},f_{D}\right)s_{r}\left(f_{ST},f_{SR},f_{D}\right)} \end{array}.$$

According to Equation (17), IF is a function of the space transmit frequency $f_{ST}$, space receive frequency $f_{SR}$, and Doppler frequency $f_{D}$. Usually, normalized frequencies are more convenient and are thus commonly used in analysis. The normalized space transmit and receive frequencies are defined as the space transmit and receive frequencies normalized to the reciprocal of the corresponding sensor spacing. The normalized Doppler frequency is defined as the Doppler frequency normalized to the pulse repetition frequency.

**Figure 3:**
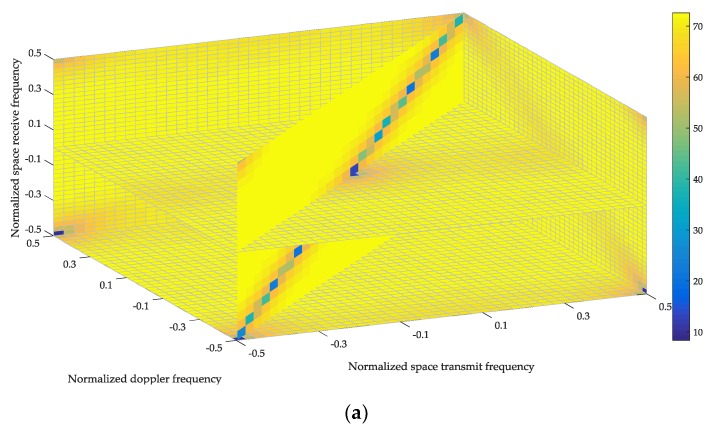

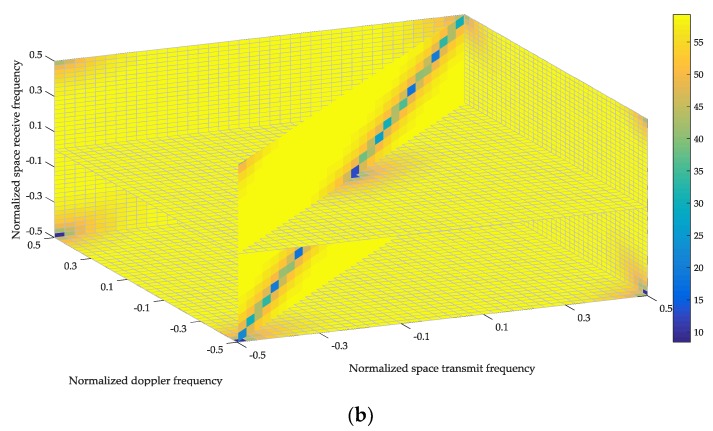
Variations of improvement factor (IF) with normalized frequencies. Results achieved by the proposed and conventional methods are shown in (**a**) and (**b**), respectively.

Figure 3 demonstrates IFs achieved by the proposed method and the conventional method. Figure 3a is the result in the second CPI. In the first CPI, the STTWs are all set equal to one, and the STRWs are determined by (13). In the second CPI, the STTWs are calculated according to (14) and STRWs are calculated according to (13), which indicates that STTWs and STRWs are updated in the iteration. Then IF is calculated by (17) and Figure 3a is generated according to IF. In Figure 3b, the IF is achieved by setting STTWs to one and calculating STRWs according to (13). It indicates that Figure 3b is the same as IF achieved by the proposed method in the first CPI.

**Figure 4:**
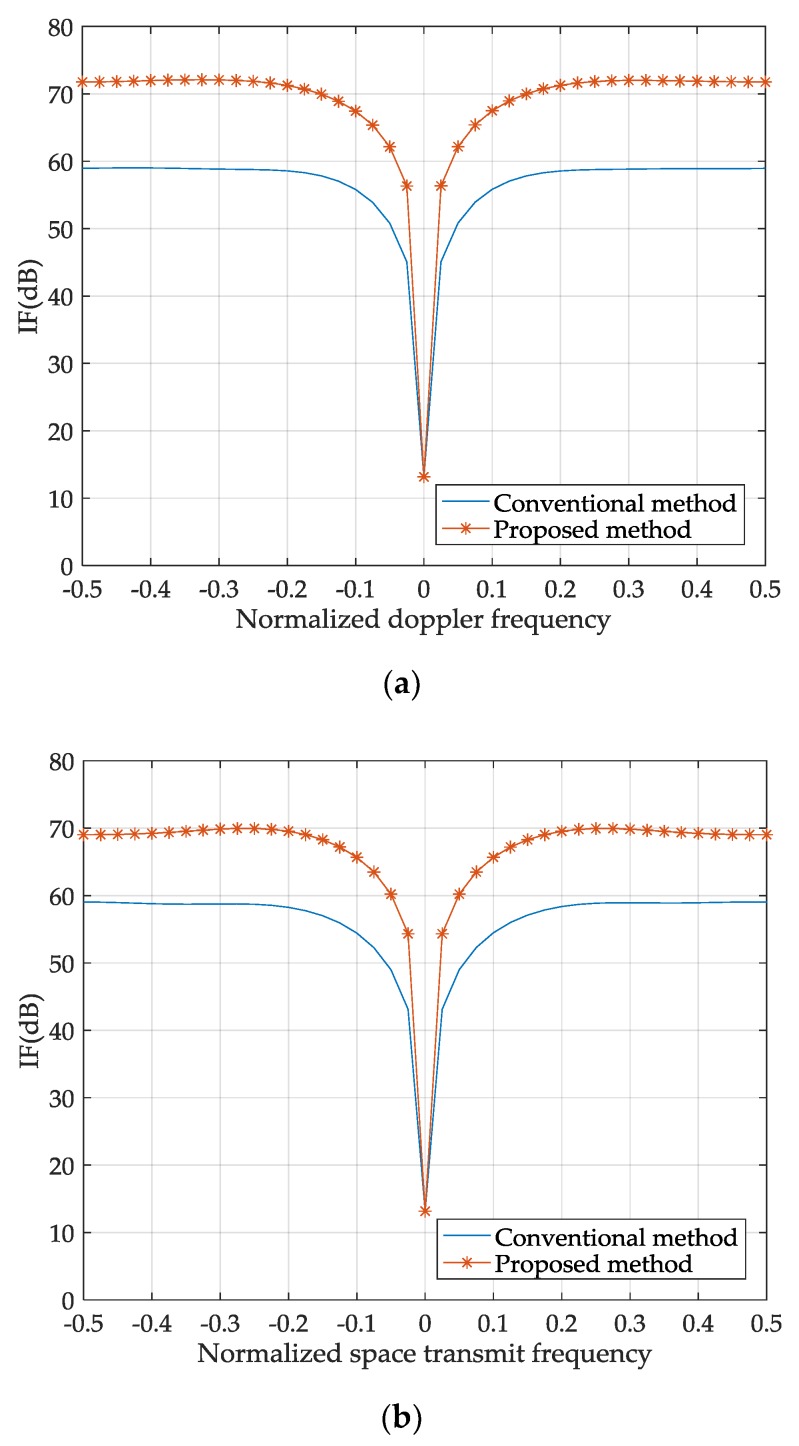

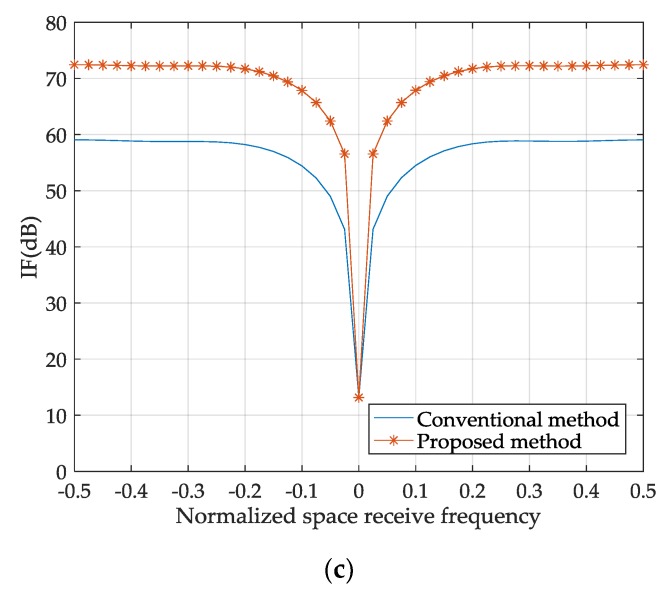
IF curves of the proposed and conventional methods. IF curves with respect to the normalized Doppler frequency, space transmit frequency, and space receive frequency are shown in (**a**), (**b**), and (**c**), respectively. In each figure, two other normalized frequencies are zero.

The distributions of IFs in Figure 3a,b are similar. In each figure, the narrow trench is formed along the clutter trajectory, which indicates that the clutter can be suppressed. Everywhere outside the clutter trench, the IF plateau exists, where detection of moving targets is optimum. The difference between the two figures is that the IF plateau of the proposed method is higher than that of the conventional method, which means that the proposed method has better performance. Figure 4 illustrates this issue more clearly by displaying three profiles of the stereograms in Figure 3. The variations of IF with normalized frequencies show that the minimums of the notches achieved by the two methods are nearly the same. Outside the notches, the IF achieved by the proposed method is more than 13 dB better than that of the conventional method.

**Figure 5:**
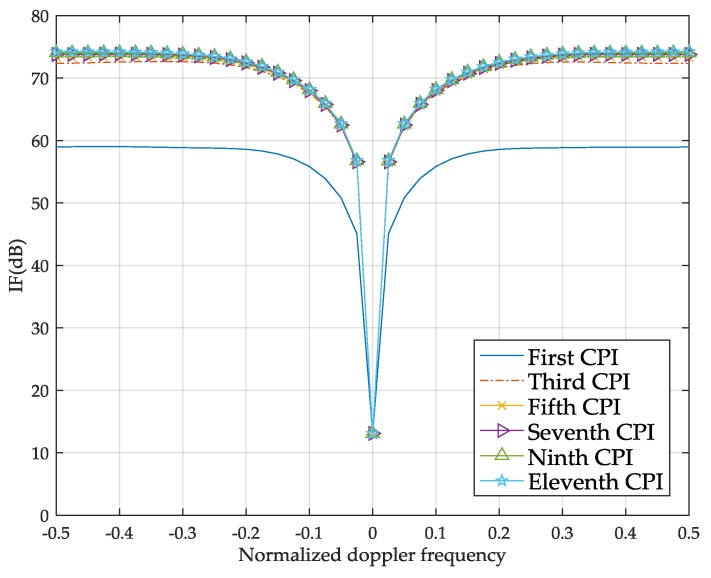
IF curves with respect to the normalized Doppler frequency.

Figure 5 demonstrates the IF curves with respect to the normalized Doppler frequency as the number of CPIs increases. Initially, the STTWs are all set to one in the first CPI. Therefore, the IF curve depending on the first CPI also represents the performance of the conventional method. Once the radar system has received echoes, the STTWs are adaptively updated by exploiting the information contained in the previous CPI. From one CPI to the next CPI, the interference-plus-noise output power is suppressed more and more, which leads to improvement of the SINR. The moving targets analyzer raises the threshold with the SINR to better extract the information of the target. Therefore, the IF curve improves as the CPI increases. Thus, a few CPIs are needed to allow the system to reach the optimal state. As shown in Figure 5, the proposed method approximately converges after the fifth CPI. Compared with the conventional method, the improvement of IF is greater than 13 dB, which is equivalent to the improvement of the output SINR under the condition that the input SINR is constant.

## 5. Conclusions

This paper has proposed a joint design of space-time transmit and receive weights. By exploiting the information of the clutter and target in the return echoes, the output SINR of colocated MIMO radar can be improved. Moreover, because of rapid convergence, the proposed approach is suitable for real-time applications. Although the proposed approach is designed for the single moving target, it can be extended to more complicated situations. Future research will focus on designing robust strategies following and accounting for target steering mismatches that can arise due to multipath and phase noise [37,38,39]. And the upper bound to the IF will also be studied.

## Figures and Tables

**Table 1:** Simulation parameters.

Parameter	Value
carrier frequency	10 GHz
pulse repetition frequency	12 KHz
inter-CPI interval	0.125 s
platform speed	90 m/s
altitude	3 km
number of transmit elements	4
number of receive elements	4
transmit sensor spacing	0.015 m
receive sensor spacing	0.015 m
target speed	56.61 m/s
target initial range	10 km
target initial direction	90°
signal/noise ratio	−10 dB
clutter/noise ratio	20 dB
number of temporal samples	8

## Appendix A

Equation (8) is rewritten as follows:

(A1) $$\underset{\omega_{t}}{\min}\omega_{r}^{H}Q_{q}\left(\omega_{t}\right)\omega_{r},\;\begin{array}{c} \begin{array}{cc} s.t. & \omega_{r}^{H}s_{r}\left(\omega_{t}\right)=1 \end{array}\\ \begin{array}{cc} & \omega_{t}^{H}s_{t}=g \end{array} \end{array}.$$

However, the objective function and the first constraint are implicit functions of $\omega_{t}$. Therefore, to obtain the solution to $\omega_{t}$, they must be changed.

The objective function equals

(A2) $$\begin{array}{cc} \underset{\omega_{t}}{\min}\omega_{r}^{H}Q_{q}\left(\omega_{t}\right)\omega_{r} & =\underset{\omega_{t}}{\min}\omega_{r}^{H}Q_{c}\left(\omega_{t}\right)\omega_{r}\\ & =\underset{\omega_{t}}{\min}\omega_{r}^{H}E[\left(\int_{\varphi =0}^{2\pi }A^{\left(c\right)}\left(\varphi \right)s_{r}^{\left(c\right)}\left(\varphi ,\omega_{t}\right)d\varphi \right)\\ & \times {\left(\int_{\varphi =0}^{2\pi }A^{\left(c\right)}\left(\varphi \right)s_{r}^{\left(c\right)}\left(\varphi ,\omega_{t}\right)d\varphi \right)}^{H}]\omega_{r}\\ & =\underset{\omega_{t}}{\min}\omega_{r}^{H}E[\left(\int_{\varphi =0}^{2\pi }A^{\left(c\right)}\left(\varphi \right)s_{r}^{\left(c\right)}\left(\varphi ,1_{MN}\right)⊙\left(1_{N}⊗\omega_{t}\right)d\varphi \right)\\ & \times {\left(\int_{\varphi =0}^{2\pi }A^{\left(c\right)}\left(\varphi \right)s_{r}^{\left(c\right)}\left(\varphi ,1_{MN}\right)⊙\left(1_{N}⊗\omega_{t}\right)d\varphi \right)}^{H}]\omega_{r}\\ & =\underset{\omega_{t}}{\min}\omega_{r}^{H}Diag\left\{1_{N}⊗\omega_{t}\right\}E[\left(\int_{\varphi =0}^{2\pi }A^{\left(c\right)}\left(\varphi \right)s_{r}^{\left(c\right)}\left(\varphi ,1_{MN}\right)d\varphi \right)\\ & \times {\left(\int_{\varphi =0}^{2\pi }A^{\left(c\right)}\left(\varphi \right)s_{r}^{\left(c\right)}\left(\varphi ,1_{MN}\right)d\varphi \right)}^{H}]Diag\left\{1_{N}⊗\omega_{t}^{*}\right\}\omega_{r}\\ & =\underset{\omega_{t}}{\min}\omega_{r}^{H}Diag\left\{1_{N}⊗\omega_{t}\right\}E\left[x_{c}\left(1_{MN}\right)x_{c}^{H}\left(1_{MN}\right)\right]Diag\left\{1_{N}⊗\omega_{t}^{*}\right\}\omega_{r}\\ & =\underset{\omega_{t}}{\min}{\left(1_{N}⊗\omega_{t}\right)}^{Τ}Diag\left\{\omega_{r}^{*}\right\}Q_{c}\left(1_{MN}\right)Diag\left\{\omega_{r}\right\}\left(1_{N}⊗\omega_{t}^{*}\right)\\ & =\underset{\omega_{t}}{\min}\omega_{t}^{Τ}Q_{c,r}\left(1_{MN}\right)\omega_{t}^{*} \end{array},$$

where

(A3) $$\begin{array}{l} Q_{c,r}\left(1_{MN}\right)=FQ_{c}\left(1_{MN}\right)F^{H}\\ F=\left[Diag\left\{\omega_{r,1}^{*}\right\},Diag\left\{\omega_{r,2}^{*}\right\},\cdots ,Diag\left\{\omega_{r,N}^{*}\right\}\right]\\ Q_{c}\left(\omega_{t}\right)=E\left[\left(\int_{\varphi =0}^{2\pi }A_{}^{\left(c\right)}s_{r}^{\left(c\right)}\left(\varphi ,\omega_{t}\right)d\varphi \right){\left(\int_{\varphi =0}^{2\pi }A_{}^{\left(c\right)}s_{r}^{\left(c\right)}\left(\varphi ,\omega_{t}\right)d\varphi \right)}^{H}\right] \end{array}.$$

The superscripts ${\left(\cdot \right)}^{Τ}$ and ${\left(\cdot \right)}^{*}$ denote the transpose and conjugate operators, respectively; $1_{N}$ and $1_{MN}$ are column vectors of all-ones with dimensions of N and MN, respectively; and Diag{a} denotes the diagonal matrix whose diagonal elements are the elements of the vector a. The STRWs vector can be expressed as $\omega_{r}={\left[\omega_{r,1}^{Τ},\omega_{r,2}^{Τ},\cdots ,\omega_{r,N}^{Τ}\right]}^{Τ}$, and $\omega_{r,i}^{}$ represents the *i*-th sub vector with the dimension of MN. Additionally, $s_{sf,i}^{}=\exp\left(j2\pi f_{s}\left(i-1\right)\right)$ represents the *i*-th element of the space steering vector.

The first constraint can be expressed as

(A4) $$\begin{array}{cc} 1 & =\omega_{r}^{H}s_{r}\left(\omega_{t}\right)\\ & =\omega_{r}^{H}\left[s_{sf}⊗\left(s_{t}⊙\omega_{t}\right)\right]\\ & =\sum_{i=1}^{N}s_{sf,i}\omega_{r,i}^{H}\left(s_{t}⊙\omega_{t}\right)\\ & =\left[\left(\sum_{i=1}^{N}s_{sf,i}\omega_{r,i}^{H}\right)⊙s_{t}^{Τ}\right]\omega_{t} \end{array}.$$

Let $s_{1}=\left[\left(\sum_{i=1}^{N}s_{sf,i}\omega_{r,i}^{*}\right)⊙s_{t}^{}\right]$ and $s_{2}=s_{t}^{*}$. Then the optimization problem becomes

(A5) $$\underset{\omega_{t}^{}}{\min}\omega_{t}^{T}Q_{c,r}\left(1_{MN}\right)\omega_{t}^{*},\;\begin{array}{c} \begin{array}{cc} s.t. & s_{1}^{H}\omega_{t}^{*}=1 \end{array}\\ \begin{array}{cc} & s_{2}^{H}\omega_{t}^{*}=g \end{array} \end{array}.$$

By using the Lagrange multiplier method, the solution is

(A6) $$\omega_{t}^{}={\left(\frac{\left[\left(D_{22}-gD_{12}\right)Q_{c,r}^{-1}\left(1_{MN}\right)s_{1}+\left(gD_{11}-D_{21}\right)Q_{c,r}^{-1}\left(1_{MN}\right)s_{2}\right]}{D_{11}D_{22}-D_{12}D_{21}}\right)}_{}^{*},$$

where

(A7) $$\begin{array}{l} D_{11}=s_{1}^{H}Q_{c,r}^{-1}\left(1_{MN}\right)s_{1}\\ D_{12}=s_{1}^{H}Q_{c,r}^{-1}\left(1_{MN}\right)s_{2}\\ D_{21}=s_{2}^{H}Q_{c,r}^{-1}\left(1_{MN}\right)s_{1}\\ D_{22}=s_{2}^{H}Q_{c,r}^{-1}\left(1_{MN}\right)s_{2} \end{array}.$$
